# Elevated Circulating Angiogenic Progenitors and White Blood Cells Are Associated with Hypoxia-Inducible Angiogenic Growth Factors in Children with Sickle Cell Disease

**DOI:** 10.1155/2012/156598

**Published:** 2012-05-23

**Authors:** Solomon F. Ofori-Acquah, Iris D. Buchanan, Ifeyinwa Osunkwo, Jerry Manlove-Simmons, Feyisayo Lawal, Alexander Quarshie, Arshed A. Quyyumi, Gary H. Gibbons, Beatrice E. Gee

**Affiliations:** ^1^Department of Pediatrics, Division of Hematology/Oncology, Emory University School of Medicine, 2015 Uppergate Dr. NE, Atlanta, GA 30322, USA; ^2^Department of Pediatrics, Morehouse School of Medicine, 720 Westview Drive, SW Atlanta, GA 30310-1495, USA; ^3^Cardiovascular Research Institute, Morehouse School of Medicine, 720 Westview Drive, SW Atlanta, GA 30310-1495, USA; ^4^Morehouse College, 830 Westview Dr SW, Atlanta, GA 30314, USA; ^5^Biostatistics Core, Morehouse School of Medicine, 720 Westview Drive, SW Atlanta, GA 30310-1495, USA; ^6^Department of Medicine, Division of Cardiology, 1462 Clifton Road N.E. Suite 507, Atlanta, GA 30322, USA

## Abstract

We studied the number and function of angiogenic progenitor cells and growth factors in children aged 5–18 years without acute illness, 43 with Hemoglobin SS and 68 with normal hemoglobin. Hemoglobin SS subjects had at least twice as many mononuclear cell colonies and more circulating progenitor cell than Control subjects. Plasma concentrations of erythropoietin, angiopoietin-2, and stromal-derived growth factor (SDF)-1**α** were significantly higher in children with Hemoglobin SS compared to Control subjects. In a multivariate analysis model, SDF-1**α** concentration was found to be associated with both CPC number and total white blood cell count in the Hemoglobin SS group, suggesting that SDF-1**α** produced by ischemic tissues plays a role in mobilizing these cells in children with Hemoglobin SS. Despite having a higher number of angiogenic progenitor cells, children with Hemoglobin SS had slower migration of cultured mononuclear cells.

## 1. Introduction

 Sickle cell anemia (Hemoglobin SS) is characterized by hemoglobin polymerization and the formation of inflexible sickled erythrocytes. Accumulation of sickled erythrocytes in the microcirculation causes acute vaso-occlusive events that lead to pain and acute organ injury. Chronic arterial vasculopathy, with intimal proliferation and arterial stenosis, can lead to complications such as stroke and pulmonary hypertension. The etiology of arterial stenosis in sickle cell anemia is poorly understood. We hypothesize that intimal proliferation in sickle cell anemia is due to abnormal reparative responses to ongoing vessel injury. Hemolytic anemia, vaso-occlusion, and abnormal flow dynamics in sickle cell anemia may contribute to vessel injury. Chronic intravascular hemolysis releases free heme, which binds avidly to nitric oxide (NO), causing NO depletion, and subsequent vaso-constriction and inflammation [1]. Erythrocyte-derived reactive iron and oxygen species are also directly injurious to endothelium [2]. Repetitive episodes of acute vaso-occlusion cause tissue ischemia and reperfusion, which also lead to inflammation and increased oxidative stress [3]. Evidence of ongoing inflammation and vascular injury is present in people with sickle cell anemia even when asymptomatic, with elevated levels of high sensitivity C-reactive protein (hsCRP) [4] and circulating endothelial cells [5].

Reendothelization after vascular injury is a critically important process to restoring and maintaining vascular homeostasis. Endothelial progenitor cells (EPCs) are recruited from the bone marrow and home to sites of vascular injury. Recruitment and homing of EPCs are intimately regulated by cytokines and growth factors released at the sites of vascular insult. Reduced numbers of endothelial progenitor colonies have been found in adults with cardiovascular risk factors [6], diabetes [7], and those with established cerebrovascular disease [8]. Cardiovascular disorders are also associated with functional impairments in EPC migration or angiogenesis [9]. Endothelial progenitor cells are elevated during acute myocardial infarction [10], stimulated by hematopoietic growth factors such as erythropoietin [11], granulocyte colony-stimulating factor (G-CSF), or granulocyte-macrophage colony stimulating factor (GM-CSF), and by treatment with HMG-CoA reductase inhibitors (statins) [12] or angiotensin-2 receptor antagonists [13].

 To date, there is limited information about the number and function of EPCs or the growth factors involved in EPC recruitment and homing in people who have sickle cell disease. Van Beem reported elevated numbers of circulating EPCs (expressing CD34 and VEGFR2) in adults with Hemoglobin SS or S*β* ^0^-thalassemia during painful crisis, but there was no difference between asymptomatic adults with sickle cell disease and healthy controls [14]. The higher number of circulating EPCs during painful crisis was associated with increased serum levels of erythropoietin, soluble VCAM-1 (sVCAM-1), and vascular endothelial growth factor (VEGF). 

Several angiogenic growth factors have been found to be elevated in Hemoglobin SS. Angiopoietin (Ang)-2 and erythropoietin were higher in adults with Hemgoglobin SS compared to healthy controls and further elevated during acute painful crisis [15]. Higher levels of vascular endothelial growth factor (VEGF) were found in subjects with Hemoglobin SS compared to controls in some studies [16, 17], but not in others [15]. When present, higher VEGF levels were found to be associated with reduced odds of elevated tricuspid valve regurgitant velocity by echocardiography in children with sickle cell disease, a noninvasive measure suggesting pulmonary artery hypertension [16]. Conversely, children with sickle cell disease with elevated tricuspid regurgitant velocity had higher concentrations of platelet-derived growth factor (PDGF)-BB. Higher levels of SDF-1 have been found in adults with Hemoglobin SS than controls, particularly in those who had pulmonary hypertension [18].

There is ongoing debate about the *in vitro* phenotype of endothelial progenitor cells. Circulating cells expressing hematopoietic stem cell marker CD34, vascular endothelial growth factor receptor (VEGFR)-2, and early progenitor marker CD133 have been considered to represent EPCs, though recent studies show that these cells were immature hematopoietic cells that did not differentiate into EPCs or form vessels [19]. In a study of the effects of granulocyte-macrophage colony-stimulating factor (GM-CSF) on vascular function in adults with peripheral arterial disease, treatment-induced increase in the number of circulating CD34-expressing cells correlated with clinical improvements in flow-mediated dilation and pain-free walking time [20], suggesting that undifferentiated hematopoietic cells have angiogenic potential or are a surrogate marker of vascular repair cells. In this paper, we refer to the cultured cells as mononuclear cells and the cells measured from the peripheral blood as circulating progenitors cells (CPCs) with angiogenic potential.

 Taken together, there is evidence that people with sickle cell disease have vessel injury and proangiogenic growth factor responses, but limited information about vascular reparative function in sickle cell disease. We hypothesize that vascular complications in people with sickle cell disease arise from altered repair mechanisms, most likely due to abnormal angiogenic cell functions. We expect CPC numbers to be normal or elevated, stimulated by high levels of erythropoietin that is seen with chronic anemia. We report here our findings of cultured mononuclear colony and CPC number in children with Hemoglobin SS versus healthy Controls, their relationship to plasma levels of angiogenic growth factors, and the migration of cultured mononuclear cells.

## 2. Materials and Methods

### 2.1. Blood Sample Collection

The study protocol was approved by the Institutional Review Boards of Morehouse School of Medicine and Children's Healthcare of Atlanta, and the Grady Hospital Research Oversight Committee. Written informed consent was obtained from each participant's parent or guardian and verbal assent from the volunteer before sample collection. Venous blood was collected from African American children aged 5–18 years old without symptoms of acute illness. *Controls* had Hemoglobin AA or AC, and sickle cell anemia subjects had *Hemoglobin SS* or Hb S-*β* ^0^ thalassemia. Children treated with hydroxyurea, recent red blood cell transfusion within the previous 90 days, or who had cardiovascular risk factors, such as overweight or obesity, cigarette smoking, diabetes, or hypertension, were excluded. Complete blood counts were performed by standard methods by the clinical laboratories used by the clinic sites.

### 2.2. Circulating Progenitor Cell Quantitation

Whole blood samples were labeled with monoclonal antibodies for FITC-conjugated anti-human CD34 (clone 8G12, 0.6 *μ*g/mL final concentration, Becton Dickinson, Franklin Lakes, NJ), PE-conjugated anti-human VEGFR2 (clone 89106, 1.2 *μ*g/mL, R&D Systems, Minneapolis, MN), PERCP-conjugated anti-human CD45 (clone 2D1, 0.6 *μ*g/mL, Becton Dickinson), APC-conjugated anti-human CD133/1 (clone AC133, 0.85 *μ*g/mL, Miltenyi Biotec Inc., Auburn, CA), and PE-Cy7 conjugated anti-human CXCR4 (clone 12G5, 0.5 *μ*g/mL, eBioscience Inc., San Diego, CA), or their isotype controls at the same concentrations, and analyzed by FACS. Thirty microliters of antibody cocktail was added to 300 *μ*L of whole blood, or 15 *μ*L of the isotype control cocktail was added to 150 *μ*L of blood. Samples were incubated in the dark for 15 minutes. Red blood cells were lysed by adding 1.5 mL of lysing solution (NH_4_Cl 0.15 M, KHCO_3_ 10 mM, EDTA 0.1 mM) into each tube. Lysis was stopped by adding 1.5 mL of staining media (phosphate-buffered saline [PBS] without Mg^++^ or Ca^++^, heat-inactivated fetal calf serum 3%, and NaN_3_ 0.1%) and mixing gently. Immediately before acquisition on a flow cytometer, 100 *μ*L of Accucheck Counting Beads (PCB100, Invitrogen) were added and mixed gently. Cells were washed twice and resuspended in 500 *μ*L of staining media. Samples were kept in the dark until run on the flow cytometer, within 4 hours of initial processing. Data are reported for cells with low (dim) CD45 expression (which excludes mature leukocytes).

### 2.3. Mononuclear Cell Colony Assay

Mononuclear cell culture was performed according to a modification of the protocol of Hill et al. [6]. Peripheral blood mononuclear cells (PBMCs) were isolated from whole blood by density fractionation and cultured on fibronectin-coated plates (Becton Dickinson) in M199 medium (Invitrogen, Carlsbad, CA) with fetal calf serum 20% (Invitrogen) and penicillin 100 U/mL and streptomycin 100 *μ*g/mL (Invitrogen), at 37°C in 5% CO_2_. The same lot of fetal calf serum was used throughout the study for all samples. After 48 hours, nonadherent cells were replated at a concentration of 10^6^ per well in fibronectin-coated plates. Media were replenished every two days. Colony-forming units (>200 micron diameter) in each well were counted 7 days after replating. The reported number of colonies per well is the average of 3 wells per subject.

### 2.4. Immunofluorescent Staining

Mononuclear cells were seeded onto fibronectin-coated four-chamber slides (Becton Dickinson) at a concentration of 2 × 10^6^ cells per chamber. Cells were grown to confluence over 14 days with media replenished every two days. Confluent monolayers were fixed with 4% paraformaldehyde in PBS. Cells were permeabilized with ice cold methanol for five minutes and blocked with goat serum 10% (Dako, Carpinteria, CA) for one hour. Cells were incubated overnight with murine primary antibodies directed against human CD31 (clone JC70A, 10 *μ*g/mL final concentration, Dako), human endothelial nitric oxide synthase (-eNOS, clone 3, 1.25 *μ*g/mL, Becton Dickinson), human CD14 (clone TUK4, 0.5 *μ*g/mL, Dako) or isotype controls at the same concentrations (mouse IgG_1_ or mouse IgG_2a_, Dako), in PBS with goat serum 2%. Cells were washed with PBS and incubated for one hour with secondary antibodies (Alexafluor (AF)-488 goat anti-mouse IgG_1_ or AF-594 goat anti-mouse IgG_2a_, Invitrogen) at 1 : 500 dilution in PBS with goat serum 2%. Slides were counterstained with DAPI nuclear stain (Invitrogen).

### 2.5. Wound Migration Assay

Mononuclear cells were grown until confluent on fibronectin-coated plates for 14 days, as described above. An aseptic linear wound was made across the mononuclear cell monolayer using sterile 20 *μ*L pipette tip. Digital photographs were taken at 0 and 24 hours after the wound was made. The wound area was measured for each time point using image analysis software (Image-Pro Plus v. 6.2 for Windows, Bethesda, MD). The difference in wound area between hours 0 and 24 was expressed as

(1) $$\text{\%Area}\;\text{migrated}\;=\frac{{\text{Area}}_{\text{Hr}\;0}-{\text{Area}}_{\text{Hr}\;24}}{{\text{Area}}_{\text{Hr}\;0}}\;\times \;100.$$

The average wound area of 3 wells was reported for each subject.

### 2.6. Angiogenic Growth Factor Assays

Quantitative enzyme-linked immuno-sorbent assay (ELISA) for human erythropoietin, angiopoietin-2, and SDF-1*α* (R&D Systems, Minneapolis, MN), and a Human Angiogenesis Assay Panel (BioRad, Hercules, CA) were used to measure plasma concentrations of growth factors. Results for erythropoietin were expressed as mIU/mL or pg/mL of plasma for the other growth factors. Each ELISA sample was run in duplicate.

### 2.7. Oxidative Stress Markers

Plasma concentrations of the redox pairs cysteine and cystine (Cys, CySS) and reduced and oxidized glutathione (GSH, GSSG) were measured by high pressure liquid chromatography (HPLC), and the redox potentials for the Cys/CySS and GSH/GSSG couples (E_h_CySS, E_h_GSSG, resp.) were calculated using the method of Jones [21]. This method includes sample preparation and storage procedures to reduce artifacts that can be caused by hemolysis or GSH thiol-disulfide exchange. Urinary 8-isoprostanes (8-iso-Prostaglandin-F_2*α*_) were measured by ELISA (Enzo LifeLife Sciences, Plymouth Meeting, PA). 

### 2.8. Classification of Variables and Data

#### 2.8.1. Outcome Variables

The main outcome variables were blood-derived mononuclear cell colonies and CPC populations. Total white blood cell (WBC) count was analyzed as an intermediate outcome variable.

#### 2.8.2. Exposure Variables

The main exposure variable was Hemoglobin Group, defined as a categorical variable with two levels, Hemoglobin SS and Control. The other exposure variables were the clinical characteristics (age, weight, sex), peripheral blood counts, angiogenic growth factors, and oxidative stress markers.

### 2.9. Statistical Analysis

Data were summarized using frequencies and percentages for categorical variables and means (and standard deviation) for continuous variables. Data that were not normally distributed were transformed using natural logarithm transformation. The antilogarithms of the means of the transformed data are reported as geometric means.

#### 2.9.1. Univariate Analyses

Simple linear regression models were fitted to determine the relationships between the number of each CPC type (outcome variables) and individual clinical variables or biomarkers (exposure variables).

#### 2.9.2. Multivariate Analyses

Bivariate linear regression models were fitted to examine the relationship between hemoglobin group SS and number of CPCs, as well as the confounding effects of the other exposure variables. Confounders were defined as those variables causing at least 25% reduction in the *β*-coefficient compared to the main exposure variable alone. Multiple linear regression analyses were then performed to examine the relationship between hemoglobin group and number of CPCs, controlling for all confounding variables.

Similar univariate and multivariate analyses were performed using WBC as an intermediate outcome. All analyses were performed using STATA Data Analysis and Statistical software (College Station, TX), and level of statistical significance was set at 0.05.

## 3. Results

 A total of 111 children were studied, 68 Controls and 43 with Hemoglobin SS. Clinical features are shown in Table 1. As expected, children with Hemoglobin SS had significantly lower hemoglobin concentration, and higher white blood cell and platelet counts than Controls. There were no significant differences in age or sex between the two groups.

### 3.1. Angiogenic Progenitor Cells

**Figure 1:**
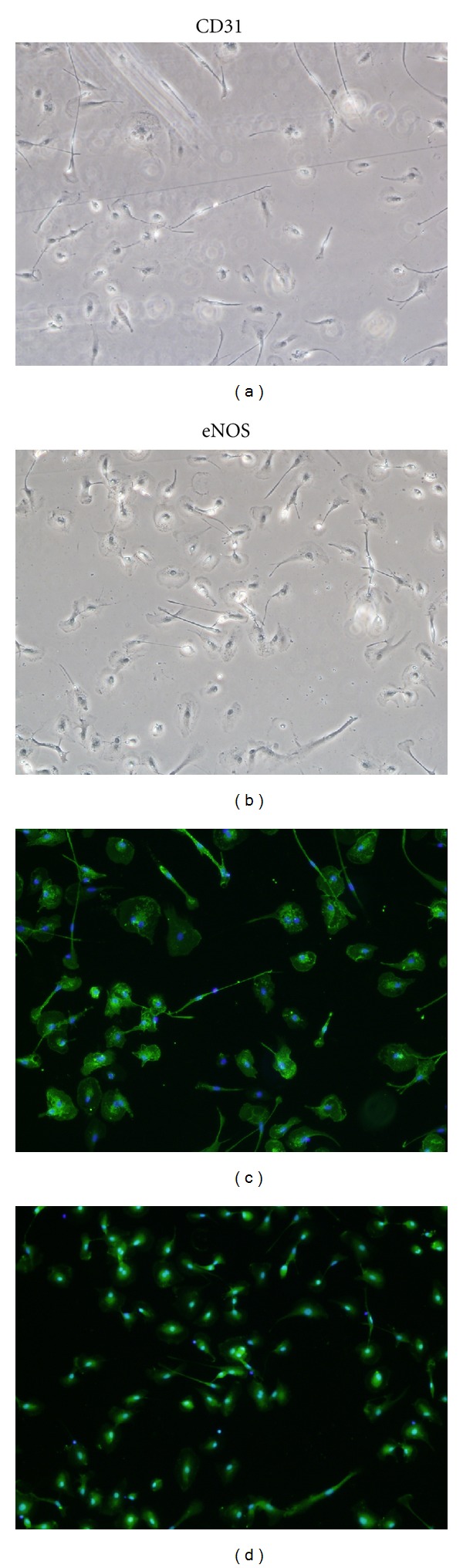
Immunofluorescent staining of cultured mononculear cells for endothelial antigens CD31 and endothelial nitric oxide synthase (eNOS). CD31 and eNOS staining was observed in a subset of cells. A representative sample from a subject with Hemoglobin SS is shown. (a) shows phase contrast image and (c) shows CD31 staining (green) with nuclei stained with DAPI (blue) in the same field. (b) Shows phase contrast and (d) shows eNOS staining (green) for the same sample.

On average, twice as many mononuclear cell colonies were grown from the blood of children with Hemoglobin SS than healthy Control children (geometric mean 16.5 versus 8.1 colonies/well, *P* < 0.05) (Table 2). A subset of the cultured cells expressed platelet endothelial cell adhesion molecule (PECAM or CD31) and endothelial nitric oxide synthase (eNOS), two markers of mature endothelial cells (Figure 1). There was no significant difference in percentage of cultured cell expressing CD31 or eNOS between Hemoglobin SS and Control groups, and the cells did not express the monocytic marker, CD14 (data not shown).

Similarly, there were at least twice as many CPCs expressing CD34, CD34/VEGFR2, CD34/CXCR4, or CD34/CXCR4/VEGFR2 in the children with Hemoglobin SS compared to Controls (Table 2), but no difference in the number of cells expressing CD34/CD133. Circulating progenitor cells expressing CD34/CD133/VEGFR2 are not reported due to very low numbers. The differences between Hemoglobin SS and Controls were highest in CPC expressing CXCR4 (SDF-1*α* receptor). Thus, using two different assay methods, we found that asymptomatic children with Hemoglobin SS have a higher number of circulating angiogenic progenitor cells.

### 3.2. Angiogenic Growth Factors

Plasma concentrations of three angiogenic growth factors were significantly higher in children with Hemoglobin SS compared to Control subjects: erythropoietin (13.5-fold), angiopoietin-2 (4-fold), and stromal derived growth factor (1.7-fold) (Table 3). In the angiogenesis multiplex assay, there were no significant differences between Hemoglobin SS and Control subjects in plasma concentrations of vascular endothelial growth factor (VEGF), hepatic growth factor (HGF), interleukin (IL)-8, follistatin, platelet endothelial cell adhesion molecule (PECAM)-1, or platelet-derived growth factor (PDGF-BB) (20 subjects tested in each group) (data not shown). The concentrations of the three elevated angiogenic growth factors were found to be collinear, which is consistent with their common regulation by hypoxia inducible factor (HIF) (data not shown).

### 3.3. Oxidative Markers

Both cysteine and cystine were significantly higher (1.5-fold) in the children with Hemoglobin SS compared to Controls. There was no difference in levels of reduced glutathione (GSH) between groups, whereas the oxidized form of glutathione (GSSG) was 2-fold lower in the children with Hemoglobin SS (Table 3). The calculated redox potential for CyS/CySS (E_h_CySS) was significantly lower (more reduced) in the Hemoglobin SS group than Controls, but there was no difference in E_h_GSSG between groups. Urinary isoprostane levels were also not different between groups.

### 3.4. Wound Migration

**Figure 2:**
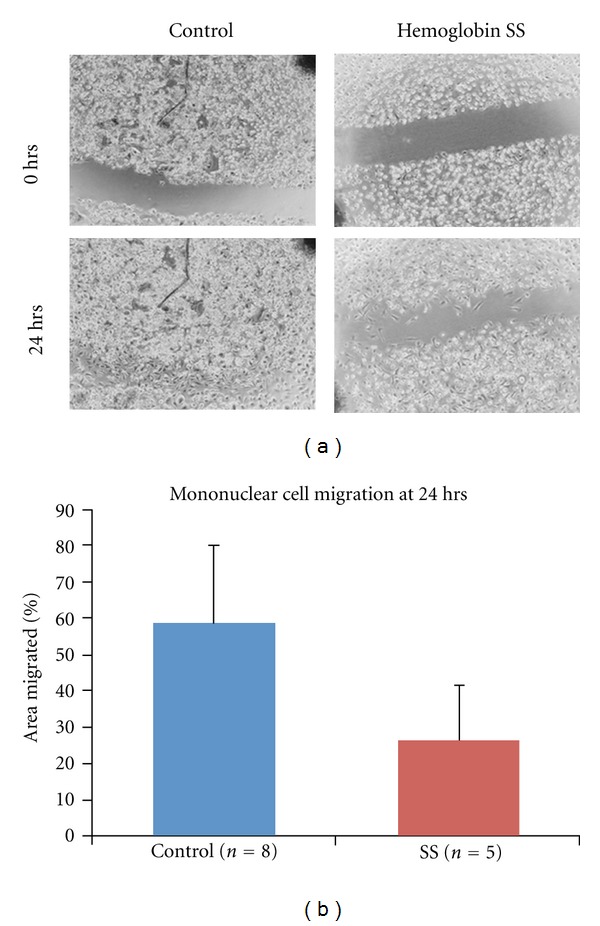
Mononuclear cell migration. Migration across a wound over 24 hours *was significantly less *in children with Hemoglobin SS than Controls. (a) shows a representative pair of Control and Hemoglobin SS wound migration assays. The freshly made wound was photographed at 0 (zero) hours, and the area migrated is measured after 24 hours. (b) shows the cumulative data for 8 Control and 5 Hemoglobin SS samples. The mean area migrated was less in children with Hemoglobin SS (28% versus 59% of the original wound area, *P* < 0.01).

Migration across a linear wound was measured in a subset of mononuclear cell samples. Mononuclear cells from Hemoglobin SS subjects (*n* = 5) migrated over a significantly smaller percentage of the original wound area in 24 hours than cells from healthy Controls (*n* = 8) (28 versus 59%, respectively, *P* < 0.01) (Figure 2). The assay was not performed if samples had not grown to confluence within the 14–16-day culture period.

### 3.5. Multivariate Analyses

Supplementary Table  1 (available online at doi:10.1155/2012/156598) shows the results of univariate analyses. Circulating progenitor cell number was significantly associated with erythropoietin, angiopoietin, SDF-1*α*, hemoglobin concentration, WBC and platelet counts, and CySS and CySH levels. The strongest associations were found with total WBC, SDF-1*α*, or erythropoietin.

A multivariate model was then used to test the role of the exposure variables as possible determinants of the elevated number of CPCs in children with Hemoglobin SS. Total white blood cell count was strongly associated with all CPC types (Table 4). When CPC number was corrected per 100 WBC, CD34/VEGFR2-expressing cells remained significantly higher in the Hemoglobin SS group than Controls (mean 0.61 versus 0.25 per 100 WBC/*μ*L, *P* = 0.04), but there were no differences for the other CPC types. To test for the effects of the angiogenic growth factors alone, WBC was excluded from the initial model.

Erythropoietin was associated with CD34 and CD34/CXCR4 numbers, and angiopoietin-2 was associated with CD34/CXCR4/VEGFR2 number. However, SDF-1*α* was consistently associated with the number of all CPC types in the Hemoglobin SS group (Table 5). SDF-1*α* in combination with either erythropoietin or angiopoietin-2 had slightly stronger associative effects. None of the other exposure variables or biomarkers, including total hemoglobin, reticulocytes, or platelet count were found to be associated with CPC number in the Hemoglobin SS group.

 White blood cells were then analyzed as a possible intermediate outcome. In univariate analysis, total WBC was found to have a similar pattern of relationships to the exposure variables as the CPCs (Supplementary Table 2). Multivariate analysis showed that each of the hypoxia-inducible angiogenic growth factors was strongly associated with the relationship between Hemoglobin SS group and elevated WBC (Table 6).

## 4. Discussion

 Vascular complications of sickle cell anemia, such as stroke and pulmonary hypertension, begin in childhood and are characterized by early development of intimal proliferation in cerebral and pulmonary arteries in the absence of cardiovascular risk factors, such as hypertension or hyperlipidemia. The mechanisms linking the primary genetic mutation in *β*-globin structure to the development of intimal proliferation and arterial stenosis are unknown. We hypothesize that sickle cell anemia is associated with abnormal vascular repair. 

We have found that children with sickle cell anemia have a pro-angiogenic phenotype, with a higher numbers of cultured mononuclear cells that express mature endothelial markers, and CPCs with angiogenic potential, and higher angiogenic growth factor levels. The higher number of CPCs in Hemoglobin SS was associated with hypoxia-inducible angiogenic growth factors, either individually or in combination. Stromal derived factor-1*α* was found to be associated with the number of all CPC types in children with Hemoglobin SS. In contrast, the severity of anemia (hemoglobin level) was not associated with CPC number. White blood cell count was found to be an intermediate outcome, responding in a similar way to the hypoxia-inducible angiogenic growth factors. When corrected for WBC, the number of CD34/VEGFR2 cells was higher in the Hemoglobin SS group, while there were no differences in the other CPC populations. This implies that the elevation in most of the CPC populations in Hemoglobin SS was a secondary effect of WBC mobilization, but that the number of circulating CD34/VEGFR2 cells was independent of elevated WBC.

 Despite a higher number of CPCs in children with Hemoglobin SS, cultured mononuclear cells in this group migrated over a smaller area in a 24-hour period, suggesting abnormal reparative function. The wound migration assay is a well-established functional assay for endothelial progenitor cells. A limitation of our study is the small number of subjects whose cells were tested in the wound migration assay. The wound assay was not performed when the cells did not form a confluent monolayer within the two-week culture period. If the cells were tested after becoming confluent over a longer culture period, we were concerned that cell senescence would contribute to variability in the results. Therefore, our results represent only those samples with better *in vitro* cell growth.

 Our findings suggest that bone marrow-derived CD34/VEGFR2 cells in asymptomatic children with Hemoglobin SS are mobilized by hypoxia-inducible angiogenic growth factors from ongoing tissue ischemia, probably due to subclinical sickle cell vaso-occlusion. We predict that numbers will be elevated during acute sickle cell complications, that there will be progressive decline in both CPC number and function with increasing age, and that those individuals with the most severe vascular complications may have impaired function. If validated as a consistent finding, impaired mononuclear cell migration may be due to alterations in SDF-1*α*-mediated CXCR4 signaling. Endothelial progenitor cells from people with coronary artery disease were found to have slower migration, reduced vascular tube formation, and less effect in restoring circulation in a rodent ischemic limb model, in association with lower SDF-1*α*-induced phosphorylation of JAK-2, a downstream target of CXCR4 [22]. If functional abnormalities in EPCs can be identified and corrected, EPCs in people with sickle cell disease can possibly be harnessed as cellular therapy to prevent or treat vasculopathy.

**Figure 3:**
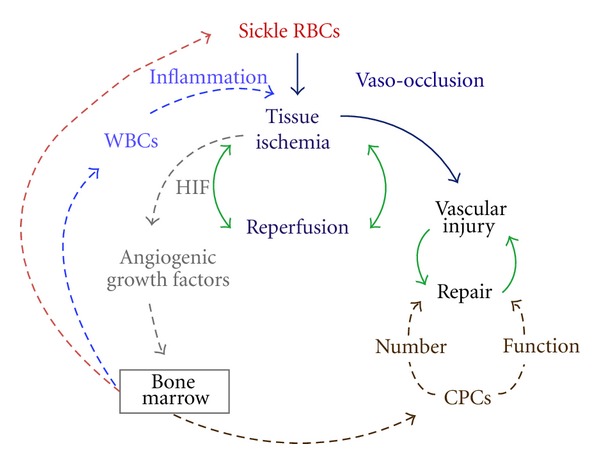
Diagram of CPC and WBC mobilization by vaso-occlusion induced tissue ischemia. Vaso-occlusion by sickled red blood cells (RBCs, red) results in tissue ischemia and reperfusion (navy blue). Repeated episodes of vascular ischemia are likely to promote vessel injury (black). Green arrows depict balanced physiologic processes that aim to restore equilibrium. Hypoxia-inducible factor (HIF, gray) produced by ischemic tissues stimulates angiogenic growth factors and bone marrow mobilization of angiogenic progenitor cells (CPCs, brown) that participate in vascular repair, white blood cells (light blue) that promote inflammation, and more sickle red blood cells.

 In addition, our data suggest that elevated WBC count in children with Hemoglobin SS is also due to mobilization by hypoxia-inducible angiogenic growth factors produced by tissue ischemia. This finding provides a mechanistic link between vaso-occlusion and the well-described elevations of white blood cells and inflammatory mediators in sickle cell disease (Figure 3). This relationship is consistent with findings from the Cooperative Study of Sickle Cell Disease (CSSCD) and a more recent cohort study, which describe elevated baseline leukocytosis as a risk factor for adverse sickle cell disease complications [23, 24]. Total WBC in the asymptomatic state, when not modified by transfusion or hydroxyurea therapy, may be a biomarker for tissue ischemia in sickle cell disease.

## 5. Conclusions

 We found angiogenic CPC number to be elevated in this group of asymptomatic children with Hemoglobin SS, while mononuclear cell migration was slower than in healthy Control children. Stromal-derived factor-1*α*, a hypoxia-inducible angiogenic growth factor, is strongly associated with the elevated numbers of CPCs and total WBC in children with Hemoglobin SS. Tissue ischemia resulting from vaso-occlusion may promote both proangiogenic and proinflammatory states in sickle cell disease.

## Figures and Tables

**Table 1:** Subject characteristics and hematologic parameters. There was no difference in age and sex of the participants in each group. Hemoglobin SS subjects had significantly lower hemoglobin concentration and higher WBC and platelet counts (indicated by asterisks (*)).

	Control			Hemoglobin SS			*P* value
	*n*	Arithmetic mean (95th% CI)	Geometric mean (95th% CI)	*n*	Arithmetic mean (95th% CI)	Geometric mean (95th% CI)	
Age (yrs)	68	12.8 (12–13.8)	12.3 (11.4–13.3)	43	12 (11–13)	11.5 (10.4–12.6)	0.26
Sex (males)	68	30 (44%)		43	20 (47%)		
Hemoglobin (gm/dL)	39	13.6 (12.1–15.2)	13.2 (12.4–14.1)	19	8.4 (7.8–9)	8.3* (7.7–8.9)	<0.001
WBC (×10^3^/mL)	39	5.4 (4.9–6)	5.2 (4.7–5.7)	18	12.1 (9.6–14.4)	11.4* (9.7–13.4)	<0.001
Platelets (×10^3^/mL)	39	280 (261–298)	274 (257–293)	18	413 (373–454)	406* (368–448)	<0.001

**Table 2:** Number of mononuclear cell colonies and CPCs for each Hemoglobin Group. Children with Hemoglobin SS had more mononuclear cell colonies and circulating progenitor cells than Controls. Mononuclear cell colonies are reported per well, and CPCs in cells/*μ*L. Significant differences in geometric means between Control and Hemoglobin SS groups are indicated by asterisks (*).

	Main exposure variables (Hemoglobin Group)							
Outcome variables	Control			Hemoglobin SS			Fold difference	*P* value
	*n*	Arithmetic mean (95th% CI)	Geometric mean (95th% CI)	*n*	Arithmetic mean (95th% CI)	Geometric mean (95th% CI)		
Colonies	63	13.5 (8.5–18.5)	8.1 (5.8–11.5)	39	28.8 (16.1–41.6)	16.5* (11–24.6)	2	0.01
CD34	43	1.7 (1.3–2.1)	1.4 (1.2–1.7)	17	5.4 (2.3–8.5)	3.6* (2.3–5.6)	2.6	<0.001
CD34/CD133	43	1.0 (0.7–1.2)	0.8 (0.7–1.0)	17	2.0 (1.0–2.9)	1.3 (0.8–2.2)		0.1
CD34/CXCR4	43	0.6 (0.5–0.7)	0.48 (0.4–0.6)	17	3.2 (1.2–5.2)	1.66* (0.9–3.2)	3.5	<0.001
CD34/VEGFR2	43	0.12 (0.09–0.15)	0.08 (0.06–0.1)	17	0.68 (0.2–1.1)	0.36* (0.2–0.6)	2.6	<0.001
CD34/CXCR4/VEGFR2	43	0.2 (0.1–0.3)	0.1 (0.07–01)	17	0.6 (0.2–1)	0.29* (0.15–0.5)	2.9	0.002

**Table 3:** Angiogenic growth factors and oxidant stress markers for each Hemoglobin Group. Children with Hemoglobin SS had higher levels of three angiogenic growth factors, higher cysteine and cystine, and lower oxidized glutathione than Controls. Significant differences in the geometric means between Control and Hemoglobin SS groups are indicated by asterisks (*).

Other exposure variables	Main exposure variables (Hemoglobin Group)							
	Control			Hemoglobin SS			Fold difference	*P* value
	*n*	Arithmetic mean (95th% CI)	Geometric mean (95th% CI)	*n*	Arithmetic mean (95th% CI)	Geometric mean (95th% CI)		
Erythropoietin (IU/mL)	68	6.6 (4.8, 8.4)	4.2 (3.2, 5.5)	42	72.4 (55.6, 89)	56.6* (43.9, 73)	13.5	<0.001
Angiopoietin-2 (pg/mL)	45	1922 (1343, 2501)	1144 (809, 1616)	27	5946 (4661, 7231)	4968* (3820, 6461)	4.3	<0.001
SDF-1*α* (pg/mL)	64	2376 (2192, 2561)	2270 (2104, 2449)	38	4065 (3660, 4471)	3877* (3491, 4305)	1.7	<0.001
CyS (*μ*M)	44	8.0 (7, 9)	7.4 (6.5, 8.4)	19	11.5 (9.5, 13.5)	10.8* (9, 13)	1.5	0.001
CySS (*μ*M)	44	23.9 (22.3, 25.5)	23.4 (21.9, 24.9)	19	34.6 (30.4, 38.8)	33.7* (30, 37.8)	1.5	<0.001
E_h_CySS (mV)	44	−81.1 (−84,–78)		18	− 87.1* (−92, −83)			0.026
GSH (*μ*M)	44	1.1 (1, 1.2)	1.0 (0.9, 1.2)	19	1.1 (0.7, 1.5)	0.89 (0.7, 1.2)		0.38
GSSG (*μ*M)	44	0.1 (0.08, 0.1)	0.08 (0.07, 0.1)	19	0.08 (0, 0.17)	0.04* (0.02, 0.07)	0.5	0.002
E_h_GSSG (mV)	44	−117.2 (−121, −113)		18	*‒*124.1 (−132, −117)			0.11
Urinary 8-isoprostanes (ng/mL)	40	15 (9.7, 20.2)	8.88 (6.2, 12.7)	16	8.9 (7.2, 10.5)	8.4 (7, 10.1)		0.83

**Table 4:** Bivariate linear regression showing relationship between Hemoglobin SS group, WBC, and CPC types. WBC was found to be a strongly associated with CPC number in the Hemoglobin SS group. Significant reductions in the beta-coefficient (>25%) compared to the main exposure variable are marked with an asterisk (*).

	Outcome variables							
Exposure variables	CD34		CD34/CXCR4		CD34/VEGFR2		CD34/CXCR4/VEGFR2	
	*β*	Adj *r* ^2^	*β*	Adj *r* ^2^	*β*	Adj *r* ^2^	*β*	Adj *r* ^2^
Group = Hb SS	0.94	0.19	1.24	0.28	1.45	0.29	1.06	0.14
Hb SS + WBC	−0.11*	0.36	0.22*	0.39	1.06*	0.29	0.32*	0.17

**Table 5:** Multivariate linear regression showing relationship between Hemoglobin SS group, angiogenic growth factors, and CPC populations. SDF-1*α* was consistently associated with the relationship between Hemoglobin SS status and all CPC types (highlighted). Significant reductions in the beta-coefficient (>25%) compared to the main exposure variable are marked with an asterisk (*).

	Outcome variables							
Exposure variables	CD34		CD34/CXCR4		CD34/VEGFR2		CD34/CXCR4/VEGFR2	
	*β*	Adj *r* ^2^	*β*	Adj *r* ^2^	*β*	Adj *r* ^2^	*β*	Adj *r* ^2^
Group = Hb SS	0.94	0.19	1.24	0.28	1.45	0.29	1.06	0.14
Hb SS + Erythropoietin	0.57*	0.16	0.51*	0.29	1.44	0.25	1.02	0.1
Hb SS + Angiopoietin-2	1.0	0.17	1.12	0.26	1.24	0.25	0.76*	0.11
Hb SS + SDF-1*α*	0.54*	0.18	0.68*	0.3	1.0*	0.27	0.37*	0.15
Hb SS + SDF-1*α* + Erythropoietin	0.42*	0.17	0.3*	0.31	1.24	0.26	0.54*	0.14
Hb SS + SDF-1*α* + Angiopoietin-2	0.71	0.19	0.73*	0.29	0.98*	0.26	0.3*	0.14

**Table 6:** Multivariate linear regression showing relationship between Hemoglobin SS group, angiogenic growth factors, and WBC. Each angiogenic growth factor was strongly associated with the relationship between Hemoglobin SS and WBC. Significant reductions in the beta-coefficient (>25%) compared to the main exposure are marked with an asterisk (*).

	Intermediate outcome variable	
Exposure variables	WBC	
	*β*	Adj *r* ^2^
Group = Hb SS	0.79	0.73
Hb SS + Erythropoietin	0.007	0.26
Hb SS + Angiopoietin-2	<0.001	0.17
Hb SS + SDF-1*α*	0.004	0.32
